# Anuran amphibian Hemoparasites over the Last Century: Advances, Challenges, and Future Prospects: A Systematic Review

**DOI:** 10.3390/ani16050847

**Published:** 2026-03-08

**Authors:** Paula Andrea Yepes, Lucas S. Barrientos, Adriana Pulido-Villamarín

**Affiliations:** 1 Unit of Ecology and Systematics (UNESIS), Department of Biology, Faculty of Sciences, Pontificia Universidad Javeriana, Bogotá 110911, Colombia; paula-yepes@javeriana.edu.co; 2 Unit of Agricultural Research (UNIDIA), Department of Microbiology, Faculty of Sciences, Pontificia Universidad Javeriana, Bogotá 110911, Colombia; adriana.pulido@javeriana.edu.co

**Keywords:** hemoparasites, amphibians, parasite prevalence, parasite diversity

## Abstract

Hemoparasites are intracellular organisms that affect groups of vertebrates, including amphibians, yet their diversity, distribution, and ecological effects remain partially understood. This systematic review analyzed advances over the past one hundred years in the study of hemoparasites in anurans, emphasizing: (1) the geographical distribution of studies, (2) the diagnostic techniques employed, and (3) the diversity of parasites and hosts involved. To this end, 83 scientific articles published between 1924 and 2024 were reviewed, following PRISMA guidelines and within an adapted PICO framework. The results reveal a notable increase in publications since the 2000s, with a concentration in North and South America. Morphological studies predominated (69.9%), while only 22.9% combined morphological and molecular techniques and 9.6% used only molecular techniques. Protozoan genera such as *Hepatozoon* and *Trypanosoma*, as well as nematodes (microfilariae) of the family Filaroidea, were frequently reported (69.7%); on the host side, the most studied anuran families were Ranidae (34.6%), Hylidae (6.9%), and Bufonidae (21.5%). Important methodological gaps were evident, particularly biodiverse tropical regions such as Central Africa, Southeast Asia, the Andean-Amazon, and Central America. These findings underscore the need to strengthen local research capacities, expand sampling efforts, and standardize diagnostic protocols to advance knowledge of hemoparasite diversity, ecology, and their role in ecosystem health.

## Simple Summary

Over the last century, scientists have reported blood parasites in frogs and toads, but we still lack a clear picture of how common they are, where they occur, and what they mean for wildlife’s health. We reviewed 83 studies published between 1924 and 2024 to bring together what is known. Scientific activity has increased since the 2000s and has been concentrated in North and South America. Most studies used microscopy to search for and identify parasites in blood smears, and far fewer combined microscopy with DNA methods that improve detection. The parasites most often reported include protozoans in the groups *Hepatozoon* and *Trypanosoma*, as well as microfilariae. On the host side, studies focused on a few Anuran amphibians. Large gaps persist in tropical regions with high biodiversity, such as parts of Africa, Asia, and the Andes–Amazon area. Filling these gaps is important because these parasites can affect the health, survival, and reproduction of amphibians, which are key to healthy ecosystems. Our work highlights practical steps—strengthening local capacities, expanding sampling, and sharing clear methods—to monitor these infections and support conservation and environmental monitoring.

## 1. Introduction

Amphibians play fundamental roles in ecosystems, serving as bioindicators of environmental change and regulating invertebrate populations [1,2]. Despite their ecological importance, amphibian populations have experienced rapid global declines, driven by habitat destruction, climate change, and the emergence of infectious diseases [3,4]. While some diseases, such as chytridiomycosis and ranavirosis, have been extensively investigated, others—such as hemoparasitic infections—have received comparatively less attention, despite their potential to increase host vulnerability [5,6].

Hemoparasitism in amphibians is caused by a broad diversity of microorganisms, including protozoa of the genera *Trypanosoma* and *Plasmodium*, as well as bacteria of the genus *Rickettsia* [7,8]. These infections can negatively affect their hosts by altering homeostasis, weakening immune responses, and compromising reproductive success and survival [9,10]. Nevertheless, research has largely focused on the taxonomic identification of parasites, often overlooking questions of prevalence, diversity, and ecological significance [11,12]. This lack of an integrative perspective has limited our understanding of the role hemoparasites may play in amphibian population dynamics and in the transmission of diseases within wild communities.

Over the past century, studies on amphibian hemoparasites have evolved from early morphological descriptions to the adoption of molecular approaches. However, most investigations continue to rely on traditional morphological techniques such as blood smear analysis, which, although useful, are limited in sensitivity and specificity [13]. These methods depend heavily on the expertise of the observer for accurate identification and for detecting immature parasite stages [12]. In recent decades, molecular tools have enabled more precise and reliable detection of these organisms [14]. Yet, their application to amphibians remains limited, and standardized protocols for cross-regional and cross-species comparisons are still lacking [6].

In light of fragmented knowledge and the absence of a comprehensive synthesis, a unified framework is needed to consolidate available information and guide future research. Here, we present a systematic review of the literature on amphibian hemoparasites spanning the past 100 years, following PRISMA guidelines [15] and using the PICO framework (Population, Intervention, Comparison, Outcome) as a structural basis. Specifically, we address three key aspects: (1) the global distribution of amphibian hemoparasite research, (2) the diagnostic methods employed and their limitations, and (3) the diversity of parasite and host species recorded. By synthesizing the state of knowledge and identifying critical gaps, this review establishes a foundation for advancing our understanding of the ecological and epidemiological roles of hemoparasites while also highlighting priorities for future research under scenarios of biodiversity loss and emerging infectious diseases.

## 2. Materials and Methods

### 2.1. Study Design

This work is a systematic review of the literature published between 1924 and 2024, following the guidelines of the PRISMA methodology (Preferred Reporting Items for Systematic Reviews and Meta-Analyses) [15]. The PICO framework (Population, Intervention, Comparison, Outcome) was used to structure the research question and to define inclusion and eligibility criteria. The population of interest (P) consisted of amphibians; the intervention (I) was the presence of hemoparasites; the comparisons (C) included diagnostic technique (morphological or molecular), publication year, and country of study; and the outcomes (O) encompassed reported prevalence, parasite types, and diagnostic approaches.

### 2.2. Search Strategy

A comprehensive literature search was conducted in three scientific databases: Web of Science, Scopus, and SciELO. The search equation was designed using Medical Subject Headings (MeSH) terms and keywords related to hemoparasites and amphibians, in English, Portuguese, and Spanish. The full search string was:

(Hemopar?sit* OR Trypanosome OR Tr*panos?oma OR “Par?sito d* la sang*e” OR “Blood parasite” OR Plasm?di* OR Rickettsia OR Filaria OR Hepatozoon OR Hemogregarin*s OR Trophozo?t* OR Microfilaria OR Apicomplexa OR Gamet* OR Dact*losoma OR Inclus* bacteri* OR Gamet?c* OR Meront* OR “Inclusi?n en globulo rojo” OR “Red blood cell inclusion” OR “Inclus?o em gl?bulo vermelho” OR Merozo? OR Intrac?toplasm?tic* OR “Inclus* citoplasm?tic*” OR “Cytoplasmic inclusion*” OR Protozo* OR Parasitemia)

AND

(Rana OR Frog OR Sapo OR Toad OR Anf?bi* OR Amphibia* OR Herpetolog* OR Salamand* OR Lagarto OR Lizard OR Bufonidae OR Lithobates OR Hylidae OR Dendrobatidae OR Leptodactylidae OR Ranidae OR Rhinella OR Phyllomedusidae)

AND NOT

(bird* OR p?jaro* OR human* OR mammal* OR mam?fero*)

The search was restricted to studies published between 1924 and 2024. Publications that did not meet inclusion criteria—such as those focused on birds, humans, or other mammals—were excluded.

### 2.3. Inclusion and Eligibility Criteria

Inclusion criteria were defined following the PICO framework (Table 1):

All types of publications (original articles, reviews, theses, and book chapters) meeting these criteria were included. Studies that did not provide methodological details or that focused on non-amphibian taxa were excluded.

### 2.4. Study Selection and Data Extraction

Records retrieved were imported into Mendeley Reference Manager (version 2.130.2) to remove duplicates. Titles and abstracts were screened to identify potentially relevant studies, and selected papers were assessed in full text for eligibility. No formal quality assessment scale was applied, as the aim of this review was to integrate all available evidence that fulfilled the inclusion criteria.

Data were extracted using a standardized template including the following variables:

- Author(s) and year of publication
- Geographic location of the study (continent and country)
- Amphibian species examined
- Hemoparasite taxa identified
- Diagnostic techniques employed (morphological or molecular)
- Reported prevalence of hemoparasitism
- Genes and primers evaluated

### 2.5. Data Analysis

Extracted data were compiled into a structured database and analyzed using descriptive and exploratory approaches. Frequencies were calculated for hemoparasite taxa, diagnostic techniques, and the geographic distribution of published studies. Temporal trends in amphibian hemoparasite research between 1924 and 2024 were assessed to identify changes in research intensity over time. Taxonomic nomenclature of amphibian hosts was updated and standardized following Frost (2023) [16]. All analyses and graphical visualizations were conducted in R (version 4.4.1), ensuring reproducibility and consistency across datasets.

## 3. Results

### 3.1. Database Search and Screening

**Figure 1:**
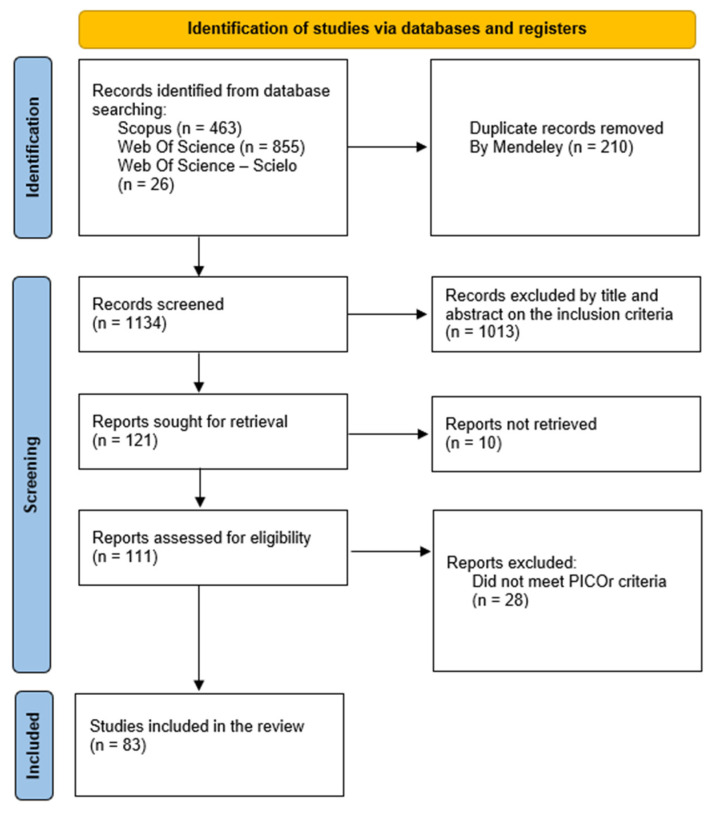
PRISMA [15] flow diagram showing the selection of Anuran amphibian hemoparasite studies between 1924 and 2024.

The systematic search in Web of Science, Scopus, and SciELO yielded a total of 1344 potentially relevant records between 1924 and 2024. The distribution of records by database is shown in Figure 1. Most records originated from Web of Science (63.6%), followed by Scopus (34.4%) and SciELO (1.9%).

### 3.2. Publications by Year and Geographic Distribution

**Figure 2:**
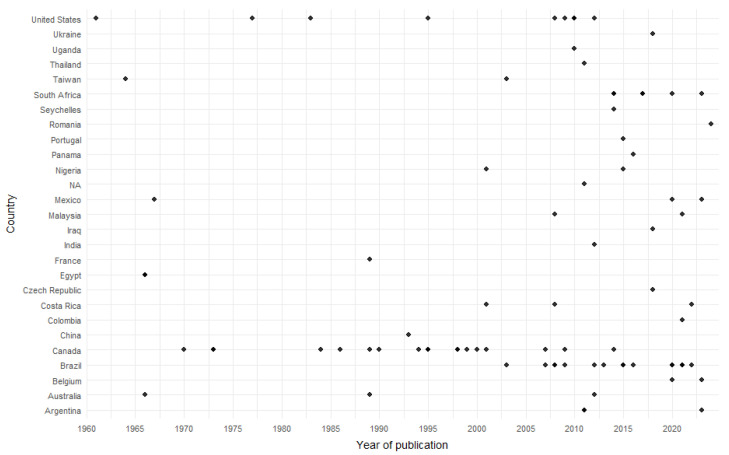
Number of publications on anuran amphibian hemoparasites per year (1960–2024).

**Figure 3:**
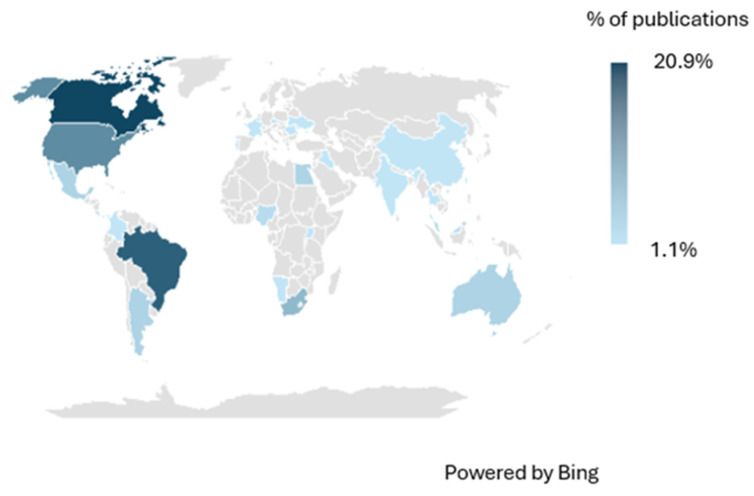
Percentage distribution of publications on Anuran amphibian hemoparasites by country over the past 100 years.

No studies were retrieved prior to 1960. From that year onwards, publications became more frequent, with clear peaks in 2008 and 2021, when five articles were published in each year (5.9%). In contrast, only one article was published in 2024, the last year analyzed. Canada accounted for the highest proportion of studies (20.9%), followed in South America by Brazil (17.6%) (Figure 2 and Figure 3) (see Supplementary Materials).

Each dot represents an individual study published in the corresponding year. Publications were identified using the PICO strategy described in the flow diagram outlining the selection process of amphibian hemoparasite studies conducted between 1924 and 2024.

### 3.3. Diagnostic Techniques

**Figure 4:**
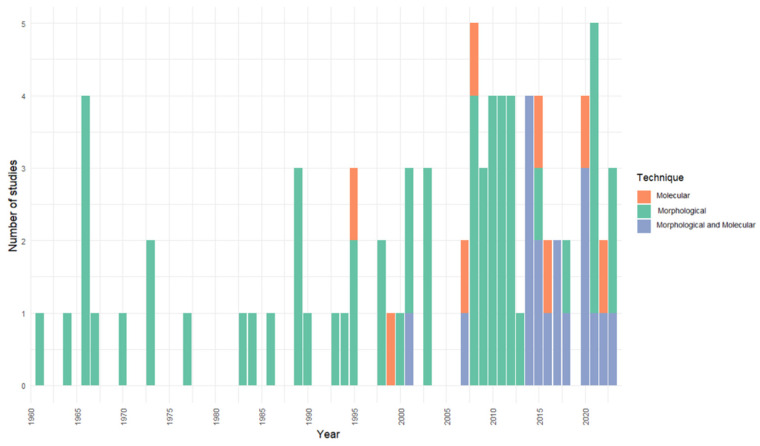
Number of studies per year according to the diagnostic technique employed (morphological, molecular, or combined).

Traditional morphological approaches predominated, being employed in 69.9% (58/83) of the studies, whereas molecular tools were used in only 9.6% (8/83). A smaller proportion of studies (22.9%; 19/83) combined both approaches. Morphological methods mainly relied on Romanowsky-based stains, particularly Giemsa, applied to blood smears fixed with methanol, followed by morphometric comparisons with previous descriptions. This enabled the identification and diagnosis of the principal hemoparasite genera reported (Figure 4). By contrast, molecular methods involved the amplification of specific DNA fragments via PCR, using primers targeting conserved regions of genes such as 18S rRNA (e.g., HepF300 and HepR900; see Supplementary Materials).

### 3.4. Hemoparasite Diversity and Amphibian Hosts

**Figure 5:**
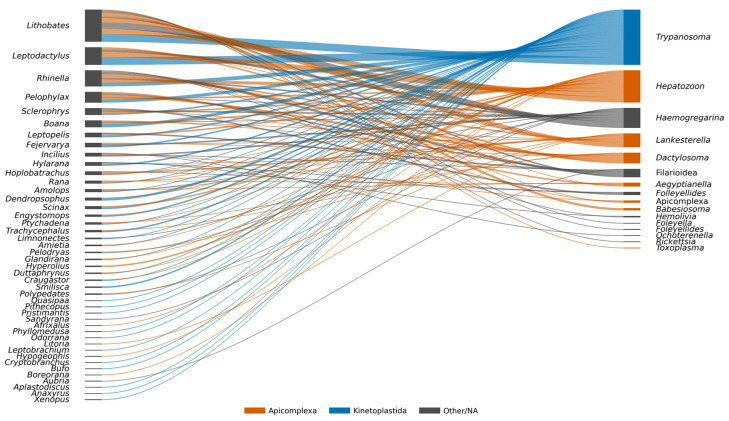
Bipartite network illustrating associations between amphibian host genera (left) and hemoparasite genera (right) reported in the literature documented between 1924 and 202. The width of each ribbon is proportional to the number of host species involved in a given association. Hemoparasite genera are color-coded according to their major taxonomic groups (Apicomplexa, Kinetoplastida, and Other/NA). Host genera are shown in black. All taxonomic names are presented at the genus level.

**Figure 6:**
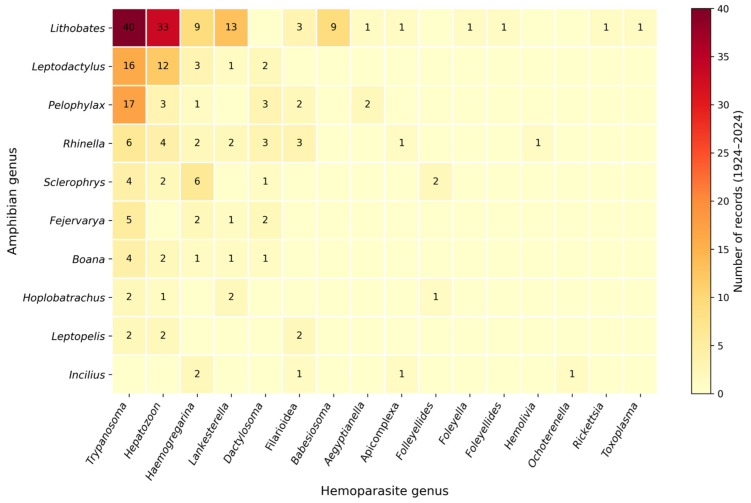
Heat map showing the frequency of reported associations between the ten most represented anuran amphibian genera and hemoparasite genera documented between 1924 and 2024. Cell values indicate the number of published records for each host–parasite association.

The most frequently reported hemoparasite genera were *Trypanosoma* (43%; 142/330), *Hepatozoon* (27.6%; 91/330), and *Lankesterella* (11.5%; 38/330). These parasites were most detected in amphibians of the genera *Lithobates*, *Leptodactylus* and *Rhinella*, which emerged as the most frequently reported hosts in the reviewed literature (Figure 5 and Figure 6). The study by Gonzales et al. (2021) [17] was not considered in this analysis because the supplementary annexes required for data extraction were unavailable (see Supplementary Materials).

To avoid confusion, it is important to clarify that this number refers to the total parasite–host associations extracted from the 83 articles included in the review. Many studies reported multiple hemoparasite species and several infected host taxa within a single publication; therefore, the number of parasite records (330) is higher than the number of articles (83). These associations formed the basis for our quantitative analyses of parasite diversity and host representation.

### 3.5. Additional Relevant Literature Published in 2025

After our systematic search had been closed (December 2024), six additional publications on amphibian blood parasites were released in 2025 and were identified [18,19,20,21,22,23] using the same search equation and scientific databases. Four of them are research articles [18,19,20,21]: Matta et al. 2025 [18] introduced the giant toad *Rhinella horribilis* as an emerging model for experimental studies of amphibian blood parasites; Votýpka et al. (2025) [19] and Sigl et al. (2025) [20] described previously unrecognized diversity and host–parasite patterns of *Trypanosoma* spp. in Neotropical frogs and in frog-biting midges (*Corethrella* spp.), respectively; and Bilhalva et al. (2025) [21] examined the potential interference of EDTA with the detection of anuran trypanosomes using Woo’s technique. In addition, two review papers were retrieved that synthesize parasite records associated with bullfrog aquaculture [22] and Egyptian amphibians [23]. Since all six articles were published after the predefined search period (1924–2024), the original dataset and analyses were not updated, and these contributions are reported here in a brief descriptive manner.

## 4. Discussion

This systematic review examined the progress made in the study of amphibian hemoparasites over the past century, focusing on three aspects: (1) the global distribution of studies, (2) the diagnostic techniques employed, and (3) the diversity of parasite and host species recorded. This approach provides a comprehensive overview of the current state of knowledge while also identifying critical gaps that must be addressed to better understand the ecological and epidemiological implications of these infections (see Supplementary Materials).

Most records retrieved were from Web of Science and Scopus, databases recognized for their broad coverage of high-impact scientific literature. In contrast, SciELO yielded relatively few studies, despite its focus on Latin American publications, which suggests a regional gap in the visibility of hemoparasite research.

The temporal analysis revealed a sharp increase in scientific interest in amphibian hemoparasites during the past two decades. Although the earliest studies date back to the mid-20th century, publication rates rose significantly after 2000, with marked growth in the 2010s. This increase is linked to advances in molecular diagnostics (including non-invasive PCR-based methods for parasite detection; [24]) and to the growing prominence of wildlife disease ecology within conservation biology [25,26,27]. Recent reviews have highlighted parasites as indicators of ecosystem health in the context of global change, reflecting this conceptual shift [28].

Beyond methodological improvements alone, the temporal increase in publications can also be understood within a broader scientific and historical context. The early 2000s marked a period of rapid technological expansion—particularly in PCR availability, sequencing platforms, and digital imaging—that transformed wildlife parasitology and enabled more precise detection of intracellular organisms. At the same time, herpetology experienced significant disciplinary growth, with expanding networks of researchers and improved natural history collections contributing to greater capacity for disease-related studies. Importantly, the global amphibian decline crisis brought unprecedented attention to amphibian health, catalyzing research programs focused on emerging infectious diseases and elevating parasites as components of conservation concern. These dynamics coincided with the rise of disease ecology and the adoption of the One Health framework, which emphasize parasites as indicators of ecosystem processes and as sentinels in wildlife–environment interactions. Increased funding opportunities targeting biodiversity loss, pathogen surveillance, and ecosystem health further stimulated research in regions with existing infrastructure. Collectively, these scientific, technological, and conceptual developments help explain the pronounced growth in hemoparasite studies observed over the last two decades.

As a result, hemoparasites have transitioned from being organisms of interest primarily to parasitologists to becoming emerging bioindicators, sensitive to factors such as climate change, habitat fragmentation, and the introduction of invasive species [29]. The increase in publications since 2008 thus reflects not only improved diagnostic capacity in terms of sensitivity and specificity but also a redefinition of hemoparasitism as an integral component of ecosystem and conservation health.

Despite this recent growth, significant geographic gaps remain. North and South America together account for more than 60% of publications, with Canada (20.9%), Brazil (17.6%), and the United States (12.1%) leading. Europe contributed approximately 17%. In contrast, highly biodiverse tropical regions—such as Central Africa, Southeast Asia, the Andes–Amazon transition, and Central America—remain underrepresented. For example, in Mexico, helminth studies cover only ~17% of amphibian species, with marked concentration in certain provinces (e.g., Veracruz, Trans-Mexican Volcanic Belt), while other regions lack any hemoparasite data [29]. Similar global biases in parasitology have been reported, with sampling effort skewed toward accessible regions with better infrastructure, leaving biogeographic corridors such as the Andes–Amazon largely overlooked [30,31]. This underrepresentation limits baseline data for monitoring emerging wildlife diseases, thereby affecting conservation and ecosystem health decision-making. However, these patterns are not merely the result of uneven sampling effort but instead reflect deeper structural and institutional limitations that shape global research capacity.

Beyond the numerical imbalance in publications, several structural factors help explain why these geographic biases persist. In many countries across Africa, Asia, and parts of Latin America, access to microscopy facilities, molecular diagnostic tools, and long-term research infrastructure remains limited, constraining the ability to detect and characterize hemoparasites with adequate resolution. These limitations are compounded by institutional barriers such as reduced funding stability, fewer specialized parasitology or wildlife disease programs, and limited laboratory capacity, all of which restrict research output despite the exceptionally high biodiversity of these regions. Language and training biases also contribute to underrepresentation: much of the available parasitological expertise is concentrated in North America and Europe, where collaborations between herpetologists and parasitologists are more established, while studies conducted in non-English-speaking countries are less frequently published in indexed journals. Moreover, the scarcity of researchers trained simultaneously in herpetology and parasitology in tropical regions results in overlooked or misidentified infections. Funding asymmetries further reinforce these disparities, as North America and Europe benefit from long-standing investment in wildlife health and disease ecology, enabling sustained sampling efforts. Finally, the prevalence of urban and peri-urban sampling—driven by proximity to universities and accessible field sites—means that remote and highly diverse ecosystems remain chronically undersampled. Together, these structural, linguistic, and institutional asymmetries offer a more complete explanation for the strong geographic biases observed in global hemoparasite literature.

From a methodological perspective, most studies rely on traditional morphological techniques. Approximately 60–70% of amphibian hemoparasite investigations were based solely on microscopy [32], while a smaller percentage used molecular tools in isolation. Only ~20% combined morphology and molecular data, yet such integrative approaches provide superior taxonomic resolution and reveal cryptic diversity that would otherwise go undetected [33].

Regarding parasite diversity, protozoans such as *Hepatozoon*, *Lankesterella*, and *Trypanosoma*, together with filarial nematodes, were the most frequently reported. Although studies on hemoparasites in other hosts (e.g., mammals, birds, reptiles) have been more common, these also tend to rely on blood smears, often limited to genus-level identification (e.g., microfilariae and *Hepatozoon* spp. in caimans and boas from Ecuador; [34]. Many amphibian records remain restricted to generic designations (“sp.”) or higher taxonomic levels, reflecting a lack of taxonomic resolution and underscoring the need for additional studies to clarify hemoparasite diversity in wild hosts.

While morphological techniques remain the “gold standard” for hemoparasite diagnosis [34], their limitations hinder robust phylogenetic analyses and restrict evaluations of ecological impacts in amphibian populations [35]. The slow adoption of molecular methods, despite their advantages in sensitivity, specificity, and phylogenetic traceability [36], may be explained by logistical, financial, or training limitations in many regions [32,33].

In terms of host representation, Ranidae, Hylidae, Bufonidae, and Leptodactylidae were the families most frequently studied, likely reflecting their broad distribution and availability as model organisms. By contrast, highly diverse families such as Craugastoridae, Strabomantidae, and Centrolenidae were underrepresented, partly due to their occurrence in tropical regions where hemoparasite research has been limited. This taxonomic and geographic bias emphasizes the need to expand sampling and characterization in poorly studied amphibian lineages, many of which may harbor undescribed parasites with important ecological or health implications.

Overall, this review highlights the urgency of promoting research in megadiverse yet underrepresented regions. This will require strengthening local capacities in parasitology and molecular biology, establishing standardized sampling and diagnostic protocols, and fostering international collaboration networks. Only through a coordinated expansion of research can existing gaps be filled, providing a more accurate picture of hemoparasite diversity and distribution while also anticipating potential impacts on ecosystem health. In countries such as Colombia—where only a handful of studies have been published over the past century—this task represents not only an academic debt but also a strategic priority for biodiversity conservation.

## 5. Conclusions

This systematic review reveals a fragmented and uneven research landscape. Most studies are concentrated in a limited number of countries in the Northern Hemisphere and South America, while vast megadiverse regions—particularly in Africa, Asia, and Central America—remain largely unexplored. Such geographic gaps limit our ability to understand global patterns of parasite diversity, host susceptibility, and emerging wildlife disease risks.

Morphological studies predominated (69.9%), while only 22.9% combined morphological and molecular techniques. Protozoan genera such as *Hepatozoon* and *Trypanosoma*, as well as nematodes (microfilariae) of the family Filaroidea, were frequently reported (69.7%). However, it is important to mention that this persistent reliance on morphological methods has restricted accurate identification of hemoparasites, limiting understanding of their diversity, ecology, and potential health implications. Although molecular tools are increasingly accessible, their implementation in amphibian hemoparasite studies remains marginal. This methodological lag underscores the urgent need for standardized protocols and strengthening local research capacities in molecular parasitology.

Host representation also remains biased. Certain anuran families have been studied more frequently due to their wide distribution and abundance, such as Ranidae 52 (34.6%), Hylidae (6.9%), and Bufonidae (21.5%), whereas endemic or range-restricted lineages—often located in tropical regions—have been systematically neglected. This omission prevents a realistic assessment of host–parasite interactions and hinders the identification of species with potential relevance or key ecological roles, as well as for identifying species with potential ecological or health relevance.

Moving forward, global research priorities should focus on (1) expanding sampling efforts in megadiverse but understudied regions; (2) implementing integrative morphological–molecular diagnostics; (3) establishing standardized and comparable parasite detection protocols; and (4) building long-term institutional capacity in parasitology and molecular diagnostics. Strengthening these priorities will improve our understanding of amphibian hemoparasitism, enhance wildlife disease surveillance, and contribute to the conservation of biodiversity, particularly in tropical ecosystems where research remains critically scarce.

## Figures and Tables

**Table 1:** PICO framework and inclusion criteria applied in the systematic review.

Component	Definition	Application in This Study
Population (P)	Target population	Amphibians (orders Anura, Caudata, and Gymnophiona)
Intervention (I)	Exposure/condition of interest	Presence of hemoparasites identified by morphological or molecular techniques ^1^
Comparison (C)	Factors of comparison	Year of publication, country/region, diagnostic method (morphological vs. molecular)
Outcomes (O)	Expected results	Reported prevalence, parasite taxa identified, diagnostic methods used

^1^ Serological studies excluded.

## Data Availability

The original contributions presented in this study are included in the article and Supplementary Materials. Further inquiries can be directed to the corresponding author.

## Supplementary Materials

The following supporting information can be downloaded at: https://www.mdpi.com/article/10.3390/ani16050847/s1, Table S1: Publications by Country, Table S2: Primers, Table S3: Amphibian Family-Hemoparasite, Table S4: Amphibian Genus-Hemoparasite, Table S5: PICO, Table S6: Bibliography screened, Table S7: PRISMA check-list, Text File S8: Graphs R code. References [7,17,32,33,37,38,39,40,41,42,43,44,45,46,47,48,49,50,51,52,53,54,55,56,57,58,59,60,61,62,63,64,65,66,67,68,69,70,71,72,73,74,75,76,77,78,79,80,81,82,83,84,85,86,87,88,89,90,91,92,93,94,95,96,97,98,99,100,101,102,103,104,105,106,107,108,109,110,111,112,113,114,115] are cited in Supplementary Materials.
