# Supplementary material for: Anuran amphibian Hemoparasites over the Last Century: Advances, Challenges, and Future Prospects: A Systematic Review
Source: Animals (Basel). 2026 Mar 8;16(5):847. doi: 10.3390/ani16050847 (PMC12984954; doi:10.3390/ani16050847)
Supplement: Supplementary file 1 [file animals-16-00847-s001.zip › Supplemental-S8_SistematicRev-Yepes.pdf]

Graphs R code

Figure 2. Number of publications on amphibian hemoparasites per year (1960–2024).

```
library(readxl) library(dplyr) library(ggplot2) library(tidyr) library(stringi) library(stringr)


# Standardize column names

nombres_originales <- names(raw) nombres_sin_acentos <-
stringi::stri_trans_general(nombres_originales, "Latin-ASCII") names(raw) <-
tolower(nombres_sin_acentos)

# Detect country and year columns

col_pais <- names(raw)[grepl("^pais$", names(raw))] col_anio <-
names(raw)[grepl("^ano$|^anio$|^fecha.*publicacion$", names(raw))]

if (length(col_pais) != 1 | length(col_anio) != 1) { stop("No pude detectar con certeza las
columnas de País y Año. Verifica que haya una columna llamada 'País' y otra 'Fecha de
Publicación'.") }

# Clean data and extract year

datos <- raw %>% rename(Pais = all_of(col_pais), Anio_raw = all_of(col_anio)) %>%
mutate(

# Extraer año de 4 dígitos aunque venga como fecha
Anio = str_extract(as.character(Anio_raw), "\\b(19\\d{2}|20\\d{2})\\b") |> as.integer()


) %>% filter(!is.na(Pais), !is.na(Anio)) %>% filter(Anio >= 1960, Anio <= 2024) %>%
mutate(Pais = factor(Pais, levels = sort(unique(Pais))))

# Scatter plot

p_scatter <- ggplot(datos, aes(x = Anio, y = Pais)) + geom_point(alpha = 0.8, size = 2) +
scale_x_continuous( breaks = seq(1960, 2024, by = 5), # ticks cada 5 años expand =
expansion(mult = 0, add = c(0, 0.8)) # espacio extra para mostrar 2024 ) +
coord_cartesian(xlim = c(1960, 2024)) + # recorta sin eliminar filas (no hay warnings)
labs( title = "", x = "Year of publication", y = "Country" ) + theme_minimal(base_size = 12) +
theme( plot.title = element_text(face = "bold"), axis.title.x = element_text(margin = margin(t
= 8)), axis.title.y = element_text(margin = margin(r = 8)) )

print(p_scatter)
```

Figure 4. Number of studies per year according to the diagnostic technique employed (morphological, molecular, or combined).

library(readxl) library(dplyr) library(tidyr) library(ggplot2) library(stringr)

#1) Function to normalize NA

to_na <- function(x) { x <- str_trim(as.character(x)) x[x %in% c("", "NA", "N/A", "na", "n/a", "-", "—", ".", "0", "No", "no")] <- NA x }

#2) Leer y estandarizar nombres (incluyendo acentos)

raw <- read_excel(ruta) |> rename(Año = matches("a(ñ|n)o", ignore.case = TRUE), Morfologica = matches("morfolog(í|i)ca", ignore.case = TRUE), Molecular = matches("^molecular$", ignore.case = TRUE)) |> mutate(Año = suppressWarnings(as.integer(Año)), Morfologica = to_na(Morfologica), Molecular = to_na(Molecular)) |> filter(!is.na(Año)) # sin año no graficamos

#3) Classification

niveles_cat <- c("Morphological", "Molecular", "Morphological and Molecular")

datos_clasificados <- raw |> mutate(Categoria = case_when(!is.na(Morfologica) & !is.na(Molecular) ~ "Morphological and molecular", !is.na(Morfologica) & is.na(Molecular) ~ "Morphological", is.na(Morfologica) & !is.na(Molecular) ~ "Molecular", TRUE ~ NA_character_), Categoria = factor(Categoria, levels = niveles_cat)) |> filter(!is.na(Categoria)) # fuera filas sin técnica

#4) Counting and Filling in Zeros

conteos3 <- datos_clasificados |> group_by(Año, Categoria) |> summarise(Conteo = n(), .groups = "drop") |> complete(Año, Categoria = niveles_cat, fill = list(Conteo = 0)) |> arrange(Año, Categoria)

#5) X-axis range according to data

min_ano <- min(conteos3$Año, na.rm = TRUE) max_ano <- max(conteos3$Año, na.rm = TRUE) inicio <- floor(min_ano / 5) * 5 fin <- ceiling(max_ano / 5) * 5

#6) Plott

ggplot(conteos3, aes(x = Año, y = Conteo, fill = Categoria)) + geom_col(position = "stack") + scale_fill_manual(limits = niveles_cat, values = c("Morphological" = "#66C2A5", "Molecular" = "#FC8D62", "Morphological and molecular" = "#8DA0CB")) + scale_x_continuous(breaks = seq(inicio, fin, by = 5), limits = c(inicio, fin), expand = c(0, 0)) + labs(title = "", x = "Year", y = "Number of studies", fill = "Technique") + theme_minimal(base_size = 12) + theme(axis.text.x = element_text(angle = 90, vjust = 0.5))

Figure 5. Bipartite network of associations between anuran families (blue circular nodes) and hemoparasite taxa identified to species or morphotype level (orange square nodes).

```
# Bipartite network with numerical labels on each edge (number of interactions)

import pandas as pd

import matplotlib.pyplot as plt

import networkx as nx

from matplotlib.lines import Line2D


# Load and prepare data

file_path = "/mnt/data/Asociaciones.xlsx"

xls = pd.ExcelFile(file_path)

raw = xls.parse('Asociaciones ').dropna(how='all')

raw.columns = [

  "ID", "Familia_Anfibio", "Genero_Anfibio", "Especie_Anfibio", "Nombre_Anfibio",

  "Familia_Parasito", "Genero_Parasito", "Especie_Parasito", "Nombre_Parasito", "Extra"

]
```

```
raw = raw[raw["Familia_Anfibio"] != "Familia"]

for c in ["Nombre_Anfibio","Genero_Anfibio","Nombre_Parasito","Genero_Parasito"]:

   raw[c] = raw[c].astype(str).str.strip()

raw["Gen_Rana"] = raw["Genero_Anfibio"]

mask_r = raw["Gen_Rana"].isna() | (raw["Gen_Rana"]=="") |
(raw["Gen_Rana"].str.lower()=="nan")

raw.loc[mask_r, "Gen_Rana"] = raw["Nombre_Anfibio"].str.split().str[0]

raw["Gen_Parasito"] = raw["Genero_Parasito"]

mask_p = raw["Gen_Parasito"].isna() | (raw["Gen_Parasito"]=="") |
(raw["Gen_Parasito"].str.lower()=="nan")

raw.loc[mask_p, "Gen_Parasito"] = raw["Nombre_Parasito"].str.split().str[0]

df = raw[["Nombre_Anfibio","Gen_Rana","Nombre_Parasito","Gen_Parasito"]].dropna()


# Counts per pair (frog genus - parasite genus)

counts = df.groupby(["Gen_Rana","Gen_Parasito"]).size().reset_index(name="n")


# Select top genera (without forcing Cryptosporidium)

top_ranas =
counts.groupby("Gen_Rana")["n"].sum().sort_values(ascending=False).head(12).index.toli
st()

top_parasitos =
counts.groupby("Gen_Parasito")["n"].sum().sort_values(ascending=False).head(12).index.
tolist()


# Construct bipartite graph

B = nx.Graph()

B.add_nodes_from(top_ranas, bipartite=0)

B.add_nodes_from(top_parasitos, bipartite=1)
```

```
# Añadir aristas con peso 'n'

for _, r in counts.iterrows():

   if (r["Gen_Rana"] in top_ranas) and (r["Gen_Parasito"] in top_parasitos):

      B.add_edge(r["Gen_Rana"], r["Gen_Parasito"], weight=int(r["n"]))

# --- Layout and sizes ---

pos = nx.spring_layout(B, k=0.7, seed=42)

edge_widths = [0.5 + 0.6*B.edges[e]["weight"] for e in B.edges()]

# Etiquetas en itálicas

labels_nodes = {n: r"$\it{" + n + "}$" for n in B.nodes()}


plt.figure(figsize=(13,10))

# Edges

nx.draw_networkx_edges(B, pos, width=edge_widths, alpha=0.5, edge_color="gray")

# Nodos

nx.draw_networkx_nodes(B, pos, nodelist=top_ranas, node_color="#1f77b4",
node_shape="o", node_size=820)

nx.draw_networkx_nodes(B, pos, nodelist=top_parasitos, node_color="#ff7f0e",
node_shape="s", node_size=720)

# Node labels

nx.draw_networkx_labels(B, pos, labels=labels_nodes, font_size=9)

# Edge labels: number of interactions

edge_labels = {(u, v): B.edges[(u, v)]["weight"] for u, v in B.edges()}

nx.draw_networkx_edge_labels(

   B, pos, edge_labels=edge_labels, font_size=8,

   bbox=dict(boxstyle="round,pad=0.2", fc="white", ec="none", alpha=0.7)

)
```

```
# legend with well-separated proxies
circle_proxy = Line2D([0], [0], marker='o', color='w', markerfacecolor='#1f77b4',
                    markersize=16, linestyle='None', label='Ranas (círculo)')
square_proxy = Line2D([0], [0], marker='s', color='w', markerfacecolor='#ff7f0e',
                    markersize=16, linestyle='None', label='Hemoparásitos (cuadrado)')
plt.legend(handles=[circle_proxy, square_proxy],
          loc='center left', bbox_to_anchor=(1.02, 0.5),
          borderaxespad=1.0, frameon=False,
          labelspacing=1.8, handletextpad=1.8, handlelength=1.8)

plt.title("Red bipartita con número de interacciones por arista", fontsize=14)
plt.axis("off")
plt.tight_layout()
plt.show()
```
